# Getting to Know Ovarian Cancer Ascites: Opportunities for Targeted Therapy-Based Translational Research

**DOI:** 10.3389/fonc.2013.00256

**Published:** 2013-09-25

**Authors:** Nuzhat Ahmed, Kaye L. Stenvers

**Affiliations:** ^1^Women’s Cancer Research Centre, Royal Women’s Hospital, Parkville, VIC, Australia; ^2^Department of Obstetrics and Gynaecology, University of Melbourne, Parkville, VIC, Australia; ^3^Reproductive Development and Cancer Laboratory, Prince Henry’s Institute for Medical Research, Melbourne, VIC, Australia; ^4^Department of Developmental Biology and Anatomy, Monash University, Melbourne, VIC, Australia

**Keywords:** ovarian carcinoma, ascites, chemoresistance, recurrence, metastasis, cytokines

## Abstract

More than one third of ovarian cancer patients present with ascites at diagnosis, and almost all have ascites at recurrence. The presence of ascites correlates with the peritoneal spread of ovarian cancer and is associated with poor disease prognosis. Malignant ascites acts as a reservoir of a complex mixture of soluble factors and cellular components which provide a pro-inflammatory and tumor-promoting microenvironment for the tumor cells. Subpopulations of these tumor cells exhibit cancer stem-like phenotypes, possess enhanced resistance to therapies and the capacity for distal metastatic spread and recurrent disease. Thus, ascites-derived malignant cells and the ascites microenvironment represent a major source of morbidity and mortality for ovarian cancer patients. This review focuses on recent advances in our understanding of the molecular, cellular, and functional characteristics of the cellular populations within ascites and discusses their contributions to ovarian cancer metastasis, chemoresistance, and recurrence. We highlight in particular recent translational findings which have used primary ascites-derived tumor cells as a tool to understand the pathogenesis of the disease, yielding new insights and targets for therapeutic manipulation.

## Introduction

Ovarian cancer has the highest mortality rate of all gynecological cancers worldwide and is frequently (>75%) diagnosed at an advanced-stage (1). As the disease is asymptomatic, early detection is difficult so that at the time of diagnosis the tumor has metastasized (FIGO stages III–IV). Even with optimal debulking surgery followed by aggressive front-line chemotherapy, which results in an 80% initial cure rate, advanced-stage disease in the majority of cases is incurable. This is due to the development of a chemoresistant disease which results in recurrence within 16–22 months and a 5-year survival rate of only ∼27% (2). More than one third of ovarian cancer patients present with malignant ascites at diagnosis; additionally, development of ascites is a fundamental part of chemoresistant and recurrent disease (2, 3). The onset and progression of ascites is associated with poor prognosis and deterioration in the quality of life of patients, as ascites can cause debilitating symptoms such as abdominal pain, early *satiety* and compromised respiratory, gastrointestinal, and urinary systems (2). In newly diagnosed ovarian cancer patients, ascites is treated by using standard treatment for the underlying disease, that is, intravenous treatment of combination of platinum and taxol-based chemotherapy. However, once the chemoresistant and recurrent features of the disease develop, management of large volumes of ascites can be a major problem, and the majority of patients are subjected to frequent *paracentesis* to temporarily relieve the symptoms. This in turn can lead to visceral and vascular injury resulting in septic complications, further complicating the treatment of the patients. In addition, ascites contains a rich tumor-friendly microenvironment which not only promotes tumor cell growth and motility (4, 5) but also results in inhibiting the response of chemotherapy (6). In short, ascites plays a major role in the progression of the advanced-stage disease, emphasizing the necessity to understand its pathophysiology and its impact on the biology of ovarian tumor cells, including its role in chemoresistance and mechanisms of tumor progression.

## Mechanism of Intraperitoneal Dissemination of Ovarian Cancer

Ovarian cancer is characterized by rapid growth and spread of intraperitoneal tumors and accumulation of ascites (1). Early metastasis in ovarian cancer occurs by direct extension of cancer growth to sites proximal to primary tumors, through a series of complex processes which involves cellular proliferation, epithelial-to-mesenchymal transition (EMT) which results in tumor cells migration to distant sites, and mesenchymal-to-epithelial transition (MET) for colonization (7, 8). The early steps of cancer progression also involve disruption of the ovarian tumor capsules and shedding of malignant cells from the primary tumors into the peritoneum where they survive as single cells or free-floating multicellular aggregates, commonly known as spheroids, in the ascites. Under this scenario, attachment and disaggregation of spheroids on mesothelial extracellular matrix (ECM) allows them to anchor as secondary lesions on pelvic organs and at a later stage, metastasize to distant organs (9, 10). Dissemination to distant sites, which carries a poor prognosis for ovarian cancer patients, has been suggested to occur via transcoelomic, lymphatic, or hematogenous routes (11, 12). Among these, metastasis through transcoelomic route is commonly observed in advanced-stage patients and is frequently associated with the production of ascites (11). The term “malignant ascites” is commonly used when the tumor fluid is tested positive for malignant cells and has a high level of lactate dehydrogenase (13, 14), suggesting that the ascites may contain tumor cells with rapid proliferative rates indicative of rapid progression of the disease. The fact that Stage 1A ovarian cancers (disease is confined to the ovary) have fewer relapses (29%) than Stage 1C (59%) (capsule has ruptured and peritoneal washings are positive for malignant cells), suggests that if the tumor can be removed before it is exposed to ascites in the peritoneum, subsequent metastatic spread, and relapses can be reduced (11, 15).

## Origin of Ascites

Under normal physiological conditions, capillary membranes of the peritoneal cavity continuously produce free fluid to keep the serosal surfaces of the peritoneal lining lubricated so that there is an easy passage of solutes between the peritoneum and the adjacent organs. Two thirds of this peritoneal fluid is reabsorbed into the lymphatic channels of the diaphragm and is propelled into the right subclavian vein by the negative intrathoracic pressure (16). In cases of disseminated intra-abdominal cancer, further increased production of peritoneal fluid is induced by the tumors due to the increased leakiness of tumor microvasculature and obstruction of the lymphatic vessels (17, 18). As a result, fluid accumulation in the peritoneal cavity exceeds fluid reabsorption, resulting in the build-up of ascites. It has been suggested that the flow of ascites currents within the peritoneal cavity dictate the routes of dissemination of ovarian cancer (11, 19). The physiological factors that drive this process are gravity, diaphragmatic pressure, organ mobility, and recesses formed by key anatomical structures (20). The three most common intra-abdominal sites of ovarian cancer metastasis are the greater omentum, right subphrenic region, and pouch of Douglas, areas which have easy access to ascites (21). Detached ovarian tumor cells either singly or in the form of multicellular spheroids primarily colonize to these distant sites under the influence of ascites flow; however, little is known about the impact of ascites flow on the heterogeneity of metastatic ovarian tumors that colonize to distant sites (20).

## Soluble Components of Ascites

Accumulation of ascites is a combined result of lymphatic obstruction, increased vascular permeability and secretions of resident tumor, and associated stromal and immune cells (11). As a result, malignant ascites constitutes a dynamic reservoir of survival factors, including cytokines, chemokines, growth factors, and ECM fragments, which individually and in a combined fashion affect tumor cell growth and progression through different cellular mechanisms (4, 5, 22, 23). A recent multiplex profiling of cytokines in the ascites obtained from 10 epithelial ovarian cancer patients has demonstrated enhanced expression of several factors including angiogenin, angiopoietin, GRO, ICAM-1, IL-6, IL-6R, IL-8, IL-10, leptin, MCP-1, MIF, NAP-2, osteoprotegerin (OPG), RANTES, TIMP-2, and urokinase plasminogen activator receptor (uPAR) (24). Among these OPG, IL-10, and leptin in the ascites of ovarian cancer patients were shown to be associated with shorter progression-free survival (24). OPG, a secreted member of tumor necrosis factor receptor (TNFR) superfamily, has been shown to bind and inhibit TRAIL-induced apoptosis of ovarian cancer cells, suggesting that ovarian tumor cells in the ascites with high expression of OPG may be able to evade TRAIL-induced cell death (25). Leptin is an adipokine produced predominantly by adipocytes and leptin-mediated signaling has been shown to promote ovarian cancer cell growth *in vitro* (26). On the other hand, IL-10 is known to inhibit T helper cell proliferation, hamper dendritic cell maturation, and inhibit T cells co-stimulatory molecules suggesting that IL-10 in ascites may help tumor cells to evade host immunological surveillance (27–29). Consistent with that, ascites-derived ovarian tumor cells have been shown to constitutively release CD95 ligand (also known as Fas ligand), which can induce apoptosis in immune cells expressing CD95 (30).

Exosomes derived from the ascites of ovarian cancer patients have been shown to impair the cytotoxic activity of peripheral blood mononuclear cells (31). Malignant ascites has been shown to also contain GD3 ganglioside, which inhibits the innate natural killer T (NKT) cell activity (32), while MUC16 expressed on the surface of ovarian cancer cells has been shown to inhibit the interaction of ovarian cancer cells with natural killer cells thus providing protection to ovarian cancer cells from host immunity (33). Additionally, correlations between the occurrence of regulatory T cells (Treg) (which inhibit tumor-specific T-cell immunity) in the ascites and reduced survival in ovarian cancer patients have been noted (11). These findings suggest that ascites contain the amenities to help tumor cells evade host immunosurveillance so that the tumor cells can avail unrestricted growth characteristics.

The concentration of inflammatory cytokines such as IL-1β, IL-6, IL-8, IL-10 was shown to be significantly higher in the ascites of ovarian cancer patients compared to that present in the serum, and correlated with poor prognosis and response to therapy (34, 35). The expression of IL-8 has been associated with increased tumorigenicity and ascites formation in animal models (36). IL-6, on the other hand, not only promotes tumor growth, migration, and invasion (34, 37, 38) but also facilitate chemoresistance (39, 40) and angiogenesis (41). In addition, high level of IL-6 in ovarian cancer ascites has been associated with shorter progression-free survival (42–44). Moreover, patients who responded to chemotherapy tended to have lower ascites IL-6 levels, compared with patients who did not respond to chemotherapy (45), suggesting that level of IL-6 in the ascites of ovarian cancer patients is an independent predictor of patient’s response to therapy.

Hepatocyte growth factor present in malignant ascites of ovarian cancer patients has been shown to stimulate the migration of ovarian cancer cells (46). Finally, lysophosphatidic acid (LPA), a bioactive phospholipid present in high levels in the ascites of ovarian cancer patients and produced by ovarian cancer cells, signals through cell surface bound G-protein dependent receptors and impose diverse affects on ovarian cancer cells which includes increased transcriptional regulation of vascular endothelial growth factor (VEGF), uPA, IL-6, and IL-8 (47, 48). Among many other functions, LPA has been shown to increase *de novo* lipid synthesis in ovarian cancer cells crucial for LPA-induced proliferation of ovarian cancer cells (49). LPA also disrupts the junctional integrity of epithelial ovarian cancer cells (50) which not only results in the metastatic dissemination of ovarian cancer cells but also results in increased membrane permeability which leads to enhanced ascites accumulation (2).

Vascular endothelial growth factor is found in abundance in the ascites of ovarian cancer patients and plays a central role in modulating the tumorigenic characteristics of ovarian cancer cells. VEGF is over expressed in ovarian tumor cells and is associated with poor prognosis (51, 52). High VEGF production from primary tumors has been reported to correlate with increased metastatic spread and worse prognosis compared to low VEGF secreting tumors (53). Retroviral enforced expression of VEGF in ovarian cancer cells has been shown to dramatically reduce the time of onset of ascites formation (54). One of the mechanisms by which VEGF modulates permeability of peritoneal membranes is by down regulation of tight junction protein claudin 5 in the peritoneal endothelial cells (55). In addition, VEGF has been shown to induce tyrosine phosphorylation of cadherin-catenin complex which results in decreased endothelial junctional strength and increased permeability (56). Several factors have been shown to influence the production of VEGF by ovarian cancer cells. These included hypoxia, LPA, tumor necrosis factor, matrix metalloproteinases, insulin-like growth factor, epidermal growth factor, platelet derived growth factor, and transforming growth factor beta (2). In line with these studies, systemic administration of the VEGF-Trap have been shown to prevent ascites accumulation and inhibit the growth of disseminated cancer in mouse models (54), suggesting that VEGF expression is crucial for ascites accumulation and ovarian cancer progression. Several agents that target VEGF have been evaluated in Phase II trials in women with recurrent ovarian cancer (57). Bevacizumab, a humanized monoclonal antibody against VEGF is currently in several Phase III studies with encouraging results (58).

## Cellular Components of Ascites

The origin and phenotype of the cells in the ascites is poorly understood. Similar to other tumor microenvironments, ascites contains a complex heterogeneous mixture of “resident” and “non-resident” cell populations, each having a defined role and connected with each other through soluble mediators, some of which have been described above. Belonging to the resident components of the ascites are tumor cells and cancer-associated fibroblasts (CAFs), to be distinguished from the non-resident populations, i.e., cells recruited from the outside the tumor microenvironment such as infiltrating macrophages/monocytes, bone marrow-derived mesenchymal stem cells (MSCs), and cytotoxic or Treg (59). Tumor cells within the ascites of ovarian cancer patients are either present as single cells or, more commonly, as aggregates of non-adherent cells, also known as spheroids (60). In this scenario, multiple (a few hundred) tumor spheroids can be seen either floating or embedded in the peritoneal cavity during primary debulking surgery (61). Some of these tumor spheroids are loosely attached to the underlying mesothelium and are detached during debulking surgery, while others are tightly attached to the peritoneum as individual small adherent tumors having independent vasculatures (61).

Neoplastic progression of ovarian carcinomas in the ascites occurs as differentiated epithelial tumors floating as tumor spheroids (62). However, it has been suggested that primary ovarian tumor cells may undergo an EMT-like process during localized invasion in the peritoneum and retain mesenchymal features in advanced tumors (8, 63). Even though the mesenchymal phenotype is central to EMT, ovarian cancer cells in ascites retain epithelial features and cell–cell contacts and are able to invade (60). Although enhanced E-cadherin expression, indicative of an epithelial cell type, has been demonstrated in the tumor cells of the ascites, especially those obtained from chemoresistant recurrent ovarian tumors (60), its expression is most commonly lost in metastasis (62). E-cadherin expressing ovarian carcinoma spheroids have been shown to adhere to and invade the surrounding mesothelium (9). Spheroids undergo reduced proliferation and have limited drug penetration resulting in decreased susceptibility to chemotherapy (64) and thereby mimic traits of cancer stem cells (CSCs)-like cells (62). Contributing to the heterogeneity of the resident ascites cells, CSCs are a population of cells that resists chemotherapy and is the source of proliferating tumor cells with progressive differentiating potential (65). These CSCs, when purified by sorting and xenografted into nude mice, have been shown to generate a significantly greater tumor burden compared to unsorted tumor cells (66, 67). On the other hand, non-resident cells within the ascites include non-cancer cells such as inflammatory cells, immature myeloid cells and activated mesothelial cells, and MSCs (which can be resident or non-resident) (68), all of which influence tumor cell behavior and response to chemotherapy (69). The resident and non-resident elements of the ascites microenvironment constantly interact with each other forming a unique tumor microenvironment (69). We discuss the non-resident cell populations within the ascites in detail below.

### Immune cells infiltrating the ascites microenvironment

Recent studies have demonstrated that immune system influences the clinical outcome of high-grade serous ovarian cancer patients (70, 71). The presence of tumor-infiltrating CD8^+^ T cells in primary tumors is associated with prolonged disease-free and overall survival of ovarian cancer patients (70, 71). In this context, the polyfunctional T-cell response of ovarian cancer patients has been shown to be disrupted by the factors in the ascites (72). Some of these factors have been discussed above, while additional factors include T cell co-stimulatory ligands B7-H4, stromal derived factor (SDF)-1, Fas ligand, and soluble IL-2 receptor (70, 71). A recent study has demonstrated that ovarian tumor T cell suppression can be alleviated by leukocyte depletion, suggesting that soluble factors secreted by leukocytes may also contribute to the suppression of T cells (73). Furthermore, a high CD4/CD8 T cell ratio in ascites was shown to be an indicator of the presence of Treg, which was associated with poor survival outcome (74). It has been reported that a high T cell/Treg ratio independently predicts increased survival (75). However, it was suggested that it is not so much the presence of Treg but in general the presence of immune responsive T cells which was observed to exert survival effects (75). In addition, reduced accumulation of CD3^+^CD56^+^ cells (natural killer or natural killer-like T cells) in the ascites was also correlated with increased platinum resistance (76). Furthermore, ascites from ovarian cancer patients containing elevated levels of IL-17 (a cytokine predominately produced by Th17 and other effector T cells) was correlated with increased overall survival (77).

In addition to above, malignant ascites contains significant numbers of activated CD163^+^ M2 type of macrophages the presence of which correlates with enhanced levels of IL-6 and IL-10 and inversely correlates with relapse-free survival period in ovarian cancer patients (78). Ascites also contains rare plasmacytoid dendritic cells (PDCs) (<0.1% of blood monocytes) (79). Activated macrophages and PDCs cells secrete CCL22 which is present in high levels in the ascites of ovarian cancer patients (80). *In vivo* treatment with monoclonal antibody to CCL22 resulted in significantly decreased Treg cell migration into tumors, suggesting that CCL22 may be contributing to the presence of Treg in ascites (80). In this context, tumor-associated PDC have been shown to induce angiogenesis *in vivo* by secreting TNF-α and IL-8 (81). In contrast, myeloid dendritic cells (MDCs) were absent from malignant ascites. MDCs derived *in vitro* suppressed angiogenesis *in vivo* through production of interleukin 12. Thus, the tumor may attract PDCs to augment angiogenesis while excluding MDCs to prevent angiogenesis inhibition, demonstrating a novel mechanism for modulating tumor neovascularization (81). In addition, myeloid-derived suppression cells (MDSCs) have been found in ovarian cancers transplanted in immune-compromised mouse models (82). These are a heterogeneous population of cells derived from immature granulocytes or monocytes released from bone marrow in response to stress induced by the tumor (83). The common functional feature of these cells is the repression of infiltrating functional T lymphocytes and natural killer cells (83). Hence, these cells critically control tumor progression but its role is yet to be identified in ovarian cancer. The above studies suggest that several factors and concerted mechanisms in the ascites create a microenvironment where cancer cells can grow unhampered.

### Stromal and mesothelial cells in the ascites microenvironment

The pro-metastatic role of inflammatory stroma has been described in the literature (84). A significantly enhanced number of CAFs has been associated with high-grade ovarian tumors compared to benign and borderline tumors (85). Abundant CAFs were associated with the occurrence of lymph node and omental metastases and increased lymphatic and microvessel densities (85). CAFs isolated from high-grade ovarian tumors facilitated more migration and invasion in ovarian cancer cell lines than those isolated from normal tissues (85). In another study, CAFs isolated from omentum were shown to be activated by ovarian tumor cells to promote ovarian cancer growth, adhesion, and invasiveness through the TGFβ1 pathway (86). Interleukin-1β secreted by ovarian tumor cells was shown to induce a p53/NFκB-mediated stromal inflammatory response to support ovarian tumorigenesis (87). A recent study has provided evidence of the inter-conversion of CAFs into MSCs required for promoting tumor growth by paracrine production of inflammatory cytokines (88). Ovarian cancer-associated MSCs have also been shown to have a greater ability to promote tumor growth compared to normal MSCs (68). This was shown to be mediated through abnormal production of BMP2. Treatment *in vitro* of ovarian cancer cell lines with recombinant BMP2 was shown to enhance the production of ALDH^+^CD133^+^ ovarian CSCs (68). In another study, the expression of HOXA9, a Müllerian-patterning gene, was shown to promote ovarian cancer growth by converting normal peritoneal fibroblasts into ovarian CAFs (89). In the same study, the expression of HOXA9 was also shown to induce normal adipose and bone marrow-derived MSCs to acquire features of CAFs by transcriptional activation of TGFβ2 mediated by the expression of CXCL12, IL-6, and VEGFA. These studies, even though not directly related to CAFs in the ascites of ovarian cancer patients implicate CAFs as an important modulator of promoting ovarian tumor growth.

In addition to CAFs, ascites contains a significant proportion of activated mesothelial cells which remain as single cells or are embedded with floating spheroids. These mesothelial cells are a major source of VEGF and LPA in ascites which have demonstrated enhanced adhesion, migration, and invasion of ovarian cancer cells *in vitro* (90). Peritoneal mesothelial cells also have an enhanced expression of SDF-1/CXCR4-dipeptidyl peptidase IV (DPPIV) which has been suggested to be involved with the re-epithelization of discarded peritoneal basement membranes after the attachment of secondary tumors on the peritoneum (91).

### Cancer stem cells in the ascites microenvironment

In recent years, many reports have described the CSC characteristics of ovarian cancer (66, 69, 92). In these models, resident cells in the ascites or primary tumors have been demonstrated to have the features of self-renewal, multi-lineage differentiation, and tumor initiation characteristics *in vivo* (93, 94). CSCs in these reports have also been demonstrated to have the ability to colonize to distant sites and to survive chemotherapy. Genetic and epigenetic mechanisms appear to be the main factors in this scenario (69). *In vitro* enrichment and propagation of CSCs are achieved by growing cells in an unattached condition in the form of “spheroids” (94–96). As one of the features of ascites-derived ovarian cancer cells is to survive in a free-floating anchorage independent condition, the highest concentration of CSCs in ovarian cancer has been proposed to reside within the free-floating tumor spheroids contained in the ascites (60, 62). In support of this notion, it has recently been demonstrated that cells within the ascites have CSC characteristics (60, 93). It has also been shown that the abundance of CSCs is more in the ascites-derived spheroids of chemoresistant and recurrent patients compared to that in the chemonaive patients (60). This may be due to the chemoresistant phenotype of ovarian CSCs in ascites which remains undetected as residual tumor cells after treatment and gradually increase in number with consecutive cycle of treatments.

Wintzell et al. (97), also reported high levels of CSCs in freshly derived ascites, in both spheroids as well as in cells existing as single-cell population, but these authors concluded that the single-cell population was more enriched in CSCs than the spheroids. Both Wintzell et al. (97), and Latifi et al. (60), showed that ascites spheroids were high expressers of E-cadherin and EpCAM and low/negative expressers of vimentin, CD44 and MMPs (MMP2 and MMP9) compared to single-cell population. In addition, Latifi et al. (60), showed that the single-cell population from ascites also have high expression of MSC markers such as CD73, CD90, CD105 as well as fibroblast surface protein (FSP), indicative of the CAF-like phenotype of single cells described by Wintzell et al. (97). However, Latifi et al. (60) found high expression of Oct4, STAT3, and CA125 in spheres and lack of expression of CA125 in the single-cell population. These observations were consistent with the lack of tumor forming ability of single cells in nude mice for as long as 20 weeks while the same number of cells collected from spheres formed tumors in nude mice within 12–14 weeks (60). These observations suggest that the tumorigenic component of ascites may exist within the spheres while single cells (potentially CAFs) may be the supporting entity, which is contrary to the conclusions of Wintzell et al. (97).

Distinct pattern of CSC marker co-expression may exist in spheres and single cells of the ascites and this needs to be explored further in future studies. High expression of Oct4 in single cells as described in Wintzell et al. (97), in contrast to high expression of Oct4 in spheres shown in Latifi et al. (60) may occur due to the differences in the separation techniques used by the two studies which may impact on the phenotypic changes in the cells. In addition, differences in the recruitment of patients in two studies may also contribute to the differences in the findings. While in Latifi et al. (60), only high-grade primary serous patients were recruited, the patient cohort in Wintzell et al. (97) contained different histologic subtypes of ovarian, Fallopian tube, and peritoneal cancers. Moreover, the expression of Oct4 was deduced at the mRNA level in Latifi et al. (60), while Western blot was used to detect the protein expression of Oct4 in Wintzell et al. (97). These differences in the approaches may contribute to the ambiguity of the Oct4 status in the spheres or single cells in the two studies. Hence, future studies on bigger cohorts of ovarian cancer patients are needed to determine if ascites spheres or single cells are the main repository of CSCs. Nevertheless, existing evidence indicates that the ascites microenvironment is a CSC-niche which facilitates processes such as EMT, inflammation, hypoxia, and angiogenesis in the resident cells which ultimately determine the function and fate of CSCs (69, 98).

## Experimental and Translational Approaches to the Study of Ascites-Derived Cells

Ascites is an indicator of poor prognosis in ovarian cancer patients, with the tumor cells within the ascites postulated to play dominant roles in metastatic spread, chemoresistance, and ultimately, the recurrence of the cancer (2, 60). Hence, a thorough understanding of the biology of the ascites microenvironment is essential for developing effective therapeutic intervention for metastatic ovarian cancer. Established ovarian cancer cell lines, often originally isolated from ascites, are readily available, immortalized, and low-cost options to assess tumor cell behavior. However, the distinct disadvantage of cell lines is their accumulation of numerous genetic and phenotypic abnormalities over years of culture which no longer accurately reflect the clinical disease (99). Ascites isolated from ovarian cancer patients represents a readily accessible source of primary cancer cells and cancer-associated cells with the potential to provide direct insights into the molecular and cellular pathophysiology of ovarian cancers as they metastasize within the peritoneal cavity. Reviewed below are some of the clinically relevant model systems which have provided novel insights into the contribution of ascites-derived cells and the ascites microenvironment to ovarian cancer tumorigenicity and the metastatic progression of the disease.

### Isolation and characterization of ascites-derived cell populations

As reviewed above, ascites contains a complex heterogeneous mixture of malignant and non-malignant cell types. Tumor cells can be isolated from ascites without mechanical or enzymatic digestion (100) and, if cultured under non-adherent conditions, retain their molecular and phenotypic profiles long-term (60). Most methods devised for the isolation and primary culture of ascites-derived cells incorporate a step to remove contaminating red blood cells, with some methods further separating cell populations based on their molecular and/or phenotypic profiles (60, 61, 93, 97, 101, 102). Notably, there have been several studies which isolated presumptive CSC populations from ascites using clonal selection (93) or FACS sorting for particular cell surface markers (101) or Hoechst dye 33342 exclusion (103). Isolated cells are characterized for their expression of stem cell markers, such as Oct4, Nanog, Bmi1, ABCG2, and then tested *in vitro* and *in vivo* for self-renewal and differentiation capabilities (104). These studies resulted in the paradigm-shifting identification of ovarian CSC populations within the ascites and the recognition of the roles CSCs play in the pathophysiology of epithelial ovarian cancer. CSCs are capable of asymmetric division which enables their own self-renewal as well as the generation of the heterogeneous differentiated cell populations that comprise the majority of the tumor mass (66, 67). When transplanted into immunodeficient mice, CSCs isolated from tumors can recapitulate the primary disease (93). Furthermore, the high rate of cancer recurrence following platinum and taxol-based chemotherapeutics is thought to be due to a failure to eradicate CSCs, which exhibit heightened chemoresistance compared to the rest of the tumor (62, 67, 105, 106). These data underscore the need to understand the central regulatory pathways critical to CSC survival in order to effectively target recurrent disease therapeutically (69).

Distinct subpopulations of ascites-derived cells have also been separated during culture on the basis of their differing phenotypes. For example, mesenchymal-like cells can be separated from epithelial tumor cells on the basis of their relative adherence to low-attachment plates (60, 107). In this method, the bulk of the ascites-derived tumor cells float as aggregates while non-tumorigenic mesenchymal cells attach to the plates (60). This method has been used to understand how the biology and molecular profile of the ascites microenvironment in patients with chemonaive and chemoresistant disease differs and how these differences relate to tumor behavior in *in vitro* and *in vivo* assays (60). Specifically, these studies demonstrated that chemotherapy treatment induces a CSC-like phenotype *in vitro* (107) which is recapitulated in primary ascites-derived ovarian cancer cells from chemoresistant patients with recurrent disease (60). These findings are supported by an independent study of ascites-derived cells, which characterized stromal progenitor cells within the ascites (101). These researchers noted that ascites from patients with recurrent and late-stage epithelial ovarian cancers contained more cells with a higher expression of stem cell markers than ascites from patients with early-stage tumors (101). Various cell isolation and culture methods have been used to access ascites cell populations, and the current data present a picture of significant intra- and inter-patient heterogeneity. Nevertheless, subpopulations of cells from ascites with CSC-like cell surface protein expression profiles and self-renewal capabilities consistently display a more aggressive metastatic, chemoresistant phenotype than cell populations lacking CSC-like features in both *in vitro* and *in vivo* xenograft models of ovarian cancer metastasis (102, 105).

These novel findings suggest the need for a thorough evaluation of the subpopulations of ascites-derived cells in association with cancer stage, patient response to chemotherapy, and overall patient survival in order to identify molecular or protein signatures within ascites subpopulations with prognostic and diagnostic significance. To this end, comprehensive gene expression assessment methods such as RNA and microRNA screens, proteomics strategies, and NextGen sequencing are being applied to the analysis of ascites-derived cells (2). A recent study demonstrated the clinical potential of one such a high-throughput, integrative approach. Using microarray and clinical data from over 1000 patients with high-grade serous epithelial ovarian cancer a novel prognostic model was developed which was based on the altered profiles of family members of lethal-7 (let-7) microRNAs (108). This study identified let-7b as the master regulator of a network of genes, with higher levels of let-7b predictive of poorer outcomes after primary chemotherapy (108). Notably, patients could be stratified on the basis of their let-7b profiles into low, intermediate, and high-risk groups which corresponded to response to front-line chemotherapy and 5-year survival rates (108). While this method was developed using publicly available gene array data sets derived from advanced ovarian cancers, adaptation of this method to the study of freshly isolated ascites-derived tumor cells from chemonaive and chemoresistant patients would represent a means for improved prediction and monitoring of patients’ response to chemotherapies.

### Functional analyses of ascites-derived cell populations

As spheroid formation within ascites is postulated to directly contribute to disease spread and to the development of chemoresistance (see above), several methods have been developed for the functional assessment of ascites-derived cells *in vitro* and *in vivo*, with the aim of mirroring various *in vivo* microenvironments as accurately as possible. The overarching aim of these studies is the development of new therapeutic approaches which specifically target particular stages of ovarian cancer metastasis, e.g., the formation or stability of spheroids within the ascites to enhance sensitivity to chemotherapeutics or the attachment and invasion of spheroids into the peritoneal lining to block colonization at distal sites.

#### In vitro modeling of spheroid formation, survival, and metastasis

Cancers which spread through the blood and lymphatic vasculature undergo repeated intravasation and extravasation through vessel walls. In contrast, during ovarian cancer metastasis, cancer cells are shed from the primary tumor into the peritoneal cavity and must survive suspended within the ascites (8, 10). To model this stage of ovarian cancer metastasis, ovarian cancer cell lines or primary ascites-derived cells are maintained under non-adherent conditions, such as in hanging-drops, in a liquid overlay, or in low-attachment culture dishes (109, 110). Under non-adherent conditions, cancer cells inherently aggregate together to form multicellular spheroids, which exhibit enhanced abilities to avoid anoikis (111). The main advantage that spheroid cultures have over monolayer cultures is that spheroid cultures more accurately model the complex three-dimensional structures assumed by ovarian cancers metastasizing within the peritoneum and recapitulate the molecular (e.g., oxygen, nutrient, metabolite) gradients found *in vivo*. Thus, multicellular spheroids cultured under non-adherent conditions which mimic the ascites more accurately predict *in vivo* behaviors and responses to therapies. For example, cancer cells grown as spheroids can be up to 100 times less sensitive to chemotherapies than the same cells cultured as monolayers, reflecting the inherent chemoresistance exhibited by metastasizing ovarian cancer spheroids in a clinical setting (109). The enhanced survival capabilities of spheroids were recently demonstrated using primary ascites-derived epithelial ovarian cancer cells (112). In this study, when grown as spheroids in non-adherent culture, ascites-derived tumor cells exhibited resistance to Myxoma-virus-mediated death despite the virus entering and replicating within the spheroids. In contrast, if the tumor cells were grown as monolayers in adherent culture or if spheroids were replated onto adherent surfaces, they exhibited sensitivity to Myxoma-virus-mediated death (112). This study has important implications for the development of treatments for advanced, metastatic ovarian cancers, underscoring the need to study the non-adherent spheroid stage of ovarian cancer metastasis in the development of new therapeutic regimens in order to ensure that new treatment options are effective against tumor spheroids floating within the ascites.

For experimental study, spheroids can be harvested freshly from ascites using centrifugation or low-attachment plates (60, 97). Harvested spheroids can be replated onto different solid surfaces or co-cultured with other peritoneal cell populations to model later stages of ovarian cancer metastasis, when multicellular spheroids attach to and invade the peritoneal lining to form a secondary tumor (113–115). In particular, co-cultures of ovarian cancer cells with specific subpopulations of the peritoneal lining and omentum, such as fibroblasts (86, 116), adipocytes (117), and mesothelial cells (115) have provided particular insights into the physical, biomechanical, and chemical interactions between invading tumor cells and the peritoneal environment in the establishment of metastatic nodules within the peritoneum. These models are growing increasingly sophisticated, with the use of primary omental and peritoneal tissue for three-dimensional organotypic models (116, 118). These studies demonstrate that peritoneal and omental fibroblasts, adipocytes, and mesothelial cells directly contribute to the pro-metastatic environment of the peritoneal cavity, releasing soluble factors into the ascites, secreting ECM components, and supplying energy reserves for the invading cancer cells.

As over 75% of ovarian cancers have already metastasized at the time of diagnosis (1), the information gained from these approaches is urgently needed in order to derive novel strategies which specifically disrupt the interactions between ovarian cancer spheroids and the peritoneal microenvironment, thereby preventing the establishment of secondary tumors. In recent years, three-dimensional spheroid culture methods have been adapted to a variety of high-throughput systems, with the aim to expediting the screening the effectiveness of therapeutic compounds and identifying the key factors underlying metastatic growth and dissemination (109, 110). Of note, a recent study has used a microfluidic platform to study the effects of the hydrodynamic forces of ascites on tumor phenotype (20). This study used several on-chip analyses [immunofluorescence for epidermal growth factor receptor (EGFR); mRNA isolation for RT-PCR; and protein isolation for biomarker quantification] to show that continuous flow induced EMT in an ovarian cancer cell line, which contributed to a more aggressively invasive phenotype. These data demonstrate yet another facet of the ascites microenvironment which contributes to the ovarian cancer metastatic process (i.e., biochemical), furthermore, this experimental approach represents a high-throughput modality in which to study the efficacy of various targeted therapies in the prevention of the establishment and growth of secondary tumors.

#### In vivo modeling of the intraperitoneal environment

A number of studies have studied the role of vascularization in ovarian cancer metastasis or verified their *in vitro* results using either subcutaneous or intraperitoneal injection of ascites-derived tumor cells into nude mice, e.g., the validation of the tumor-repopulating abilities of isolated ascites-derived tumor cells or putative ovarian cancer CSCs *in vivo* (60, 93, 102, 105). These models provide an *in vivo* microenvironment for testing established and novel chemotherapeutic approaches and are a necessary preclinical model system. However, these models lack the true metastatic features of ovarian cancer which occurs in the peritoneum and involves the ovaries, adjacent organs (extra-ovarian pelvic organs, e.g., colon, bladder, liver) as well as spheroids carried around in the ascites to distal organs of the peritoneal cavity (1). Moreover, the xenotransplantation immunocompromized mouse model currently used may select populations of tumor cells that can override the weak immunogenic response of nude mice which is entirely different from the immune response in patients against their own tumors (119) In recognition of this latter problem, recently a refined mouse xenograft model has been developed using human embryonic stem cells to generate a “human” microenvironment within immunocompromized mice. Using malignant cells freshly isolated from the ascites of an ovarian cancer patient, six derivative cell subpopulations were developed, and it was found that the human microenvironment permitted some patient-derived ascites cells to generate tumors which failed to grow in a conventional nude mouse model (120). This improved method may enable the study of the *in vivo* behaviors of previously unstudied cell subpopulations and also provides insights into the role of the human microenvironment in the tumorigenicity and metastatic capabilities of ovarian cancers.

## Ascites as a Platform for Translational Research

As discussed above, ascites is a source of tumor material from which valuable information can be extracted not only to understand the pathophysiology of ovarian cancer progression but also for the development of markers which will predict prognosis and monitor the progression of the disease. The frequent presence of ascites at first presentation, and subsequent relapses, provides an accessible pool of tumor material that can be studied to determine the molecular characteristics of cells as the disease progresses. With the establishment of methods which can separate the different soluble and cellular components of the ascites (60), it may now be possible to identify and differentiate the true molecular perturbations that exist between the chemonaive, chemoresistant, and recurrent status of the disease. Isolated cellular components of the ascites can be preserved as paraffin embedded blocks for immunohistochemical analysis (121, 122), or can be frozen for molecular analysis at the RNA and protein levels (60, 122). Moreover, ascites provides a substantial amount of biological material which can be obtained to design studies which require relatively larger amounts of tumor material, which previously were only limited to genome-based studies due to the scarce availability of primary and metastatic tumors leftover after pathological diagnosis. These studies include methods to elucidate the protein profile of ascites-derived tumor and associated cells by proteomic methods such as matrix-assisted laser desorption and ionization (MALDI), surface enhanced laser desorption and ionization (SELDI), and liquid chromatography followed by mass spectroscopy (MS) (2, 123), all of which require larger amounts of samples than that used by genomic methods. In addition, high-throughput automated array-based proteomics techniques such as reverse phase protein arrays (RPAs) can be used to understand the differential expression of proteins in the isolated ascites cellular components from chemonaive and chemoresistant patients. A recent study which used the RPA analysis on ascites samples and pleural effusions obtained from ovarian cancer patients showed significantly higher expression of AKT, cAMP-responsive element binding protein (CREB), and Jun-N-terminal kinase (JNK) in malignant ascites compared to benign effusions (124). Given that deregulation of PI3 kinase and the downstream AKT pathway has been demonstrated in ovarian cancer (125, 126), and high levels of p38 and an increase in the ratio of phosphorylated EGFR and phosphorylated JNK were associated with bad prognosis in ovarian cancer patients (124), it seems that the proteomic profile of the ascites environment may imitate the protein expression profile of the original tumors (2). These observations suggest the enormous potential of using ascites samples for diagnostic, prognostic, and therapeutic endpoints.

Accessibility to ascites also provides a means of comparing the secretory components of the chemonaive and chemoresistant patients. A recent study has determined the cytokine expression profile of the ascites of ovarian cancer patients. Out of 120 cytokines analyzed OPG, IL-10, and leptin was found to be associated with worst prognosis in ovarian cancer patients (24). The concept that the damage of tumor cells in response to chemotherapy treatment can activate autocrine and paracrine secretory responses of residual tumor cells (69, 127, 128), suggest that the soluble component of the ascites microenvironment of chemonaive and chemoresistant patients may be significantly different. In addition, the tumor growth promoting effect of exosomes released by ovarian tumors has been reported (129). Malignant ascites-derived exosomes of ovarian carcinoma patients have been shown to contain CD24 and EpCAM (130). The exosome-associated proteolytic activity in the tumor vicinity has been suggested to augment tumor invasion into the stroma (130). Exosomes released by ovarian cancer cells have been shown to induce apoptosis of mature dendritic cells and peripheral blood nuclear cells suggesting they have a negative effect on host immunity (31). In addition, ascites have been shown to contain pro-survival factors which compromised the therapeutic effects of TRAIL and were shown to be associated with shorter disease-free intervals in ovarian cancer patients (131). These data suggest that the signals derived from the soluble ascites microenvironment plays a crucial role in regulating ovarian tumor cells and targeting the survival promoting activity of the soluble component of ascites may be mandatory for the development of efficient therapies for ovarian cancer patients.

## Future Directions

From a clinical perspective, our understanding of ascites and its associated cellular and soluble components are of utmost importance to understand the advanced-stage disease. The central component of such investigations would be ascites obtained from patients pre- and post-chemotherapy and understanding both the soluble and cellular components individually and/or in association with each other. These studies can be performed using microfluidic systems to investigate the impact of ascites on resident and non-resident cell systems either individually or in combination (20). Microfluidic platforms have been used to investigate the morphological parameters and migratory potentials of immune cells in response to external stimulus (132). Other studies have used cell-on-chip based platforms to investigate the interaction of tumor cells with endothelial cells (133). Recently, a simple cell-on-chip platform was developed to investigate the crosstalk between immune cells and cancer (89). Using this approach, which consisted of three wide parallel chambers interconnected via an array of short and narrow capillary migration channels, it was possible to visualize under the microscope the interaction between the immune and cancer cells (89). Hence, customized microfluidic platforms may be helpful to study and mimic the events of ascites-derived microenvironment. This can also provide helpful clinical information as understanding the crosstalk between cancer cells with associated surrounding cells in the native ascites microenvironment will result in the improvement of therapies for ovarian cancer.

## Conclusion

The accessibility of ascites undeniably provides a rich source of tumor samples to monitor the course of chemotherapy treatment in patients. In addition, it also provides an opportunity for the identification of prognostic and treatment-monitor markers, as well as options for molecular profiling of both the cellular and soluble components. The cellular and molecular profile of individual ascites is a subject of inter-patient variations which will differ not only with the treatment protocol but also how each patient responds to a particular therapy. Hence, to provide a molecular characterization which would fit into a defined pattern to design appropriate targeted therapies would be challenging. Hence, long-term, longitudinal studies within the same patient cohorts, starting with chemonaive status and periodic evaluations of molecular and cellular characterization of the ascites components as the disease progresses would be useful to develop an individualized predictive profile which will be crucial for designing targeted therapies. The interrogation of soluble and cellular variations in ascites during the treatment regimen in patients may guide clinical decision making for patient management (134). This may form a basis for informed and effective personalized treatment approaches. Hence, with the advances in our understanding of the pathophysiology of ascites and the development of new methods which can delineate the cross talk between the different cellular components it is anticipated that more effective and targeted strategies for the management of ascites and ovarian cancer patients will be available in near future.

## Conflict of Interest Statement

The authors declare that the research was conducted in the absence of any commercial or financial relationships that could be construed as a potential conflict of interest.
